# Association of GGCX gene polymorphism with warfarin dose in atrial fibrillation population in Xinjiang

**DOI:** 10.1186/1476-511X-12-149

**Published:** 2013-10-23

**Authors:** Xiayizha Kamali, Muhuyati Wulasihan, Yu-Chun Yang, Wu-Hong Lu, Zhi-Qiang Liu, Peng-Yi He

**Affiliations:** 1 Department of Cardiology, First Affiliated Hospital of Xinjiang Medical University, Urumqi 830011, P.R. China

**Keywords:** GGCX gene, Atrial fibrillation, Gene polymorphism, Warfarin

## Abstract

**Objective:**

To study the effects of γ-glutamyl carboxylase (GGCX) rs2592551 polymorphism on warfarin dose in atrial fibrillation patients in Xinjiang region.

**Methods:**

Polymerase chain reaction - restriction fragment length polymorphism and direct sequencing methods were used to detect the rs2592551 genotype in 269 atrial fibrillation patients with warfarin administration. The effects of different genotypes on warfarin dose were statistically analyzed.

**Results:**

The rs2592551 polymorphism detection results were 136 cases of wild-type homozygous CC genotype (50.56%), 115 cases of heterozygous CT genotype (42.75%), 18 cases of homozygous TT genotype (6.69%). The allele frequency C was 71.93%, T was 28.07%. The stable warfarin dose average was 2.86 ± 0.61 mg/d in patients with CC genotype, 3.59 ± 0.93 mg/d in patients with CT genotype and 4.06 ± 0.88 mg/d in patients with TT genotype. The warfarin dose in different genotypes were compared, there was statistically significant difference between CC and TT, CC and CT (*P* <0. 05), but the TT and CT showed no significant difference (P > 0.05).

**Conclusion:**

In atrial fibrillation population in Xinjiang, patients with CT and TT genotypes in GGCX gene rs259251 loci required for significantly higher warfarin dose than those with CC genotype. Therefore, rs2592551 polymorphism may one of the factors affecting the warfarin dose in patients with atrial fibrillation.

## Introduction

Atrial fibrillation (AF) is the most common sustained clinical arrhythmia, the overall incidence of atrial fibrillation was 0.4%. The incidence of atrial fibrillation increased with age, it can reach up to 10% in people over 75 years. The increased prevalence of atrial fibrillation was also associated with increased prevalence of coronary heart disease, hypertension, heart failure and other diseases. In the next 50 years, the atrial fibrillation will become one of the most popular cardiovascular disease.

Thromboembolism, particular the cerebral embolism was currently the complication with highest morbidity and mortality caused by atrial fibrillation. A retrospective survey of the hospitalized cases of atrial fibrillation in some areas showed that the prevalence of stroke was 17.5% in patients with atrial fibrillation [1]. Many studies showed that the ischemic stroke and complications will greatly increase in atrial fibrillation patients who did not receive the anticoagulant therapy. Therefore, in order to reduce the risk of stroke in atrial fibrillation patients, the timely active anticoagulant was essential in clinical treatment [2].

During atrial fibrillation, the systolic function loss and long-term heart rate increaseing will lead to heart failure and increased mortality to 2 times than healthy people [3]. There were racial differences in atrial fibrillation causes and risk factors according to the previous study [4,5]. γ-glutamyl carboxylase (GGCX) was the major enzyme affecting the metabolism of warfarin. Warfarin is the most commonly used anticoagulant in clinical treatment, and GGCX is one of the candidate genes in individual differences of response to warfarin [6-10]. In this study, polymerase chain reaction, restriction fragment length polymorphism and direct sequencing were used to genotype the rs2592551 polymorphism in GGCX gene to, analyze the relation between gene polymorphism and warfarin dose in patients with atrial fibrillation.

## Results and discussion

The distribution of GGCX gene rs2592551 was in line with Hardy-Weinberg equilibrium (*P* > 0.05).

In general clinical data comparison: Age, smoking, and blood urea nitrogen showed significant differences between men and women (*P* <0.05, Table 1), and the other indicators showed no difference (*P* > 0.05).

**Table 1:** Comparison of the clinical data between male and female

**Parameters**	**Men**	**Women**	**χ**^**2**^**or** ***t***	***P*** **value**
Age (Year)	59.03 ± 14.34	54.60 ± 14.81	2.48	<0.05
Smoking (n,%)	51(33.77)	24(20.33)	5.95	<0.05
Alcohol (n,%)	31(20.53)	15(12.71)	2.86	0.10
BMI (kg/m2 )	24.09 ± 3.08	24.35 ± 3.13	−0.69	0.50
Height (Cm)	166.34 ± 7.64	165.86 ± 8.37	0.48	0.63
Weight (kg)	69.41 ± 13.56	67.40 ± 12.58	1.24	0.22
Hypertension (n,%)	84(55.63)	61(51.69)	0.41	0.54
CAD (n,%)	87(57.62)	64(54.24)	0.31	0.62
DM (n,%)	83(54.97)	55(46.61)	1.85	0.18
SBP (n,%)	119.34 ± 18.79	116.99 ± 14.70	1.12	0.27
DBP (n,%)	71.40 ± 11.32	73.01 ± 10.84	−1.18	0.24
TG (n,%)	1.41 ± 0.98	1.28 ± 0.70	1.16	0.25
TC (n,%)	4.10 ± 0.96	4.23 ± 1.04	−1.04	0.30
HDL-C (n,%)	0.97 ± 0.31	0.94 ± 0.30	0.62	0.54
LDL-C (n,%)	2.46 ± 0.79	2.49 ± 0.73	−0.41	0.68
FBG (n,%)	6.07 ± 1.97	5.95 ± 1.95	0.48	0.64
BUN (n,%)	342.59 ± 109.03	303.69 ± 86.08	3.18	<0.00

(BMI: body mass index; SBP: systolic blood pressure; DBP: diastolic blood pressure; TG: triglycerides; TC: total cholesterol; HDL-C: high-density lipoprotein; LDL-C: low-density lipoprotein; FBG: fasting blood glucose; BUN: blood urea nitrogen).

As shown in Table 2, There was statistically significant difference between CC and TT, CC and CT (*P* <0. 05) in warfarin dose, but there was not difference between TT and CT genotype (*P* > 0. 05).

**Table 2:** Genotype frequencies, warfarin dose and its comparison in rs2592551 of GGCX gene

**N (269)**		**N (%)**	**Warfarin dose (mg/d)**	**U value**	**P value**
rs2592551 genotype	CC	136(50.56)	2.86 ± 0.61		
	CT	115(42.75)	3.59 ± 0.93		
	TT	18(6.69)	4.06 ± 0.88		
	CC vs TT			353.00	<0.000
	CT vs TT			746.00	0.057
	CC vs CT			4232.00	<0.000
	C	387(71.93)			
	T	151(28.07)			

γ-glutamyl carboxylase (GGCX) can catalyze the γ-carboxylating effects of clotting factor II, VII, IX, X. Its role was dependent on vitamin K and Oxygen (O_2_) [11]. When reduced vitamin K and O_2_ existed, GGCX incorporated CO_2_ specifically into glutamic acid residues, the γ-carboxylated glutamic acid were produced. The vitamin K 2, 3 - epoxide was also produced [12,13]. Glutamic acid residue containing vitamin K-dependent clotting factor II, VII, IX, X, protein C and protein S should be carboxylated by GGCX to acquire the activity. Then a series of cascades happened to cause blood coagulation. Therefore, GGCX was major enzyme affecting the metabolism of warfarin [14-20].

Atrial fibrillation is the most common arrhythmia in clinical practice, which can cause the high morbidity and mortality, but the cause and pathogenesis are still unclear [21].

Xinjiang is a multi-ethnic co-populated area, and the main ethnic groups are Han, Uygur, Kazak, Hui, Kirgiz, Mongolian, Tajik, etc. Due to geographical conditions, living, eating habits in various ethnic groups and genetic background in Xinjiang, there were obvious differences between these minor ethnicities and Han population in other areas. Up to date, although several studies have been reported the relation between GGCX gene polymorphisms and warfarin dose, there was no study reporting the effect of GGCX gene polymorphisms on Warfarin dose in atrial fibrillation patients in Xinjiang. The previous studies indicated that GGCX gene polymorphism had different degrees of impact on warfarin dose. It has been considered as the major genetic factor influencing warfarin dose.

In this study, we found the stable warfarin dose average was 2.86 ± 0.61 mg/d in patients with CC genotype, 3.59 ± 0.93 mg/d in patients with CT genotype and 4.06 ± 0.88 mg/d in patients with TT genotype. This results indicated that the TT genotype carriers in atrial fibrillation population in Xinjiang required higher dose of warfarin than patients with CC genotype, which was to say, the full mutation genotype required more warfarin than the wild homozygotes genotype. The CT and TT genotypes required warfarin dose which had no significant different. There was no significant difference on warfarin dose between heterozygous (part mutant) genotype and full mutation genotype.

In conclusion, GGCX gene polymorphism may be one of the factors affecting the dose of warfarin in patients with atrial fibrillation in Xinjiang. However, the results need to be further confirmed in a larger sample and different populations.

## Subjects and methods

### Subjects

From January 2009 to December 2012, the inpatients and outpatients with atrial fibrillation were selected in the First Affiliated Hospital of Xinjiang Medical University. The atrial fibrillation was confirmed by ECG or Holter monitoring, the patients with familial history, valvular heart disease, congenital heart disease and lung disease were excluded. 269 atrial fibrillation patients including 131 Han population, 83 of Kazak, 53 of Uygur, 1 of Kirgiz, 1 of Mongolian were enrolled. 118 patients were female with age from 25 to 89 years (54.60 ± 14.81) And 151 patients were male with age from 22 to 89 years (59.03 ± 14.34). Clinical data included age, gender, height, weight, recent warfarin dosage, International normalized ratio (INR) values, and drug administration.

Stable warfarin dose: With the same dose, the 2 consecutive INR values in patients were between 1.8 ~ 3.0, the separated time between 2 times of detection should be at least more than 7 d [21]. All patients did not have bleeding or embolic symptoms.

Exclusion criteria: The patients with blood diseases, coagulation disorders, liver and kidney dysfunction, severe infection, severe heart failure, anemia, cancer and drugs or food administration which had effects on warfarin doses were exclude from this study. The protocol was approved by Medical Ethics Committee of First Affiliated Hospital of Xinjiang medical university and all the patients signed written informed consent.

## Methods

### Clinical data

The height, weight were measured by specialized personnel, past history and living habits were obtained through questionnaires. Body mass index (BMI) = weight / height^2^ (kg/m^2^).

### Blood biochemical indicators tests

Serum total cholesterol (TC), high density lipoprotein cholesterol (HDL-C), low density lipoprotein cholesterol (LDL-C), triglycerides (TG), uric acid, fasting blood glucose (FBG), blood urea nitrogen, serum creatinine and other biochemical indicators were measured by inspection center of the First affiliated hospital of Xinjiang Medical University. All blood samples were taken in the morning fasting state.

### DNA extraction and PCR amplification

5 ml of fasting venous blood were obtained. After adequate anticoagulation, they were stored at −80°C. The DNA was extracted by using the whole blood genome extraction kit (TIANGEN Ltd.) Primers were designed using Primer premier 5.0 software and were synthesized by Shanghai Sangon Biological Engineering Co., Ltd. The primers sequence was shown in Table 3. PCR amplification was performed in C1000TM Thermal CyclerPCR instrument. 20 μl of PCR reaction system contained 10 μl of PCR Master mix (2×), 0.5 pmol of upstream and downstream primers, 7.5 μl of ddH_2_O, and 2 μl of genomic DNA. Cycling parameters were as follows: 95°C denaturation for 3 min, 95°C deformation for 30 s, 61.2°C annealing for 30 s, 72°C extension for 1 min, a total of 35 cycles were include, total extension at 72°C for 5 min.

**Table 3:** The loci, primer sequences, annealing temperature and the product length of amplified gene

**Gene locus**	**Primer sequences (5′-3′)**	**Annealing temperature (°C)**	**Product length (bp)**
rs2592551	GGACTTAGAAAGGAACGGATGA	61.2	381
	CTTGAGAAAAGGCAAAGCAGAC		

### Genotyping

**Figure 1:**
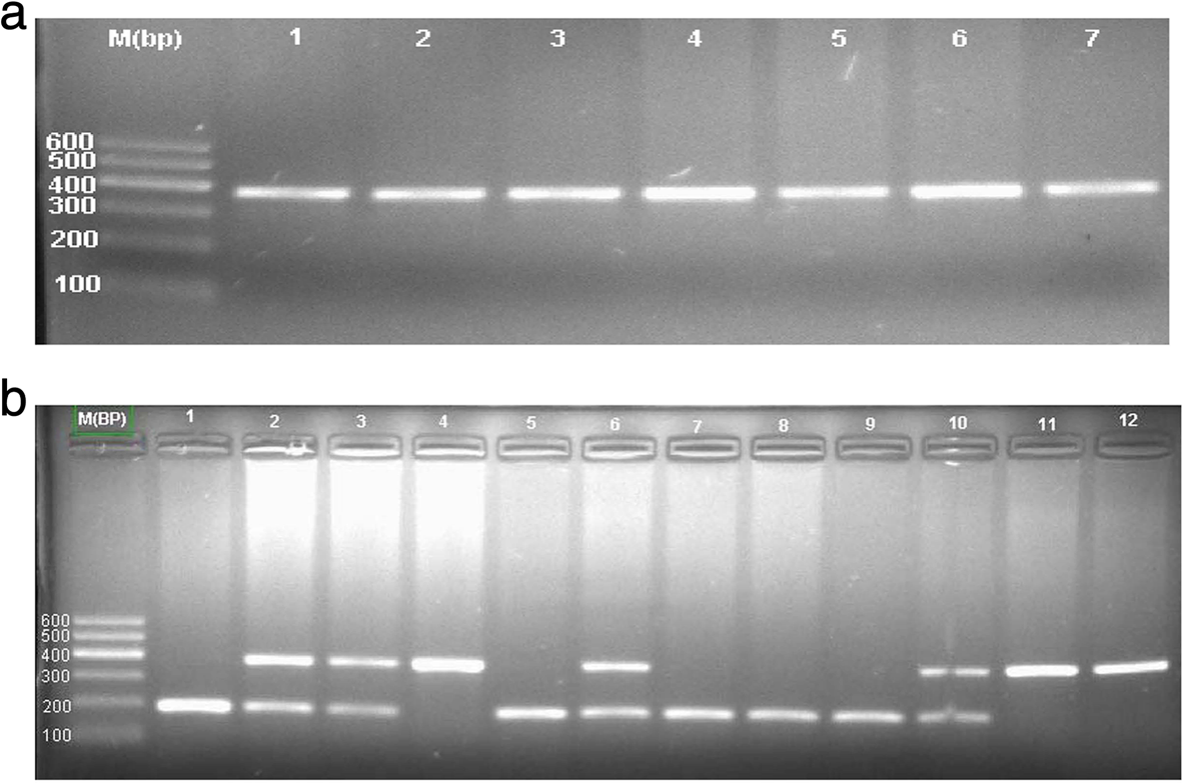
**The PCR and digestion products of rs2592551. a**: rs2592551 PCR product with product length of 381 bp; **b**: rs2592551 digestion products. 2, 3, 6, 10 were heterozygous CT genotype. 1, 5, 7, 8, 9 were mutated homozygous TT genotype. 4, 7, 8, 9 were homozygous CC wild-type (Due to the 189 and 192 bp enzyme digestion lengths were very close to each other, there showed overlap on the electropherogram showing as one band).

Polymerase chain reaction-restriction fragment length polymorphism and direct sequencing methods were used to genotype. The rs2592551 changed from C to T and one MbiI (BsrBI) restriction endonuclease was created. After Mbi digestion, there were two fragments of 189 bp and 192 bp. C allele had no restriction sites and remained a 381 bp fragment. Therefore, the CC genotype was wild type homozygous and was only one band with fragment length of 381 bp. CT genotype was heterozygous mutation, which was part mutation and had three fragments of 381,189 and 192 bp, respectively. Because the 189 bp and 192 bp was very close and the bands were overlapped each other, we only can see that CT heterozygous had two fragments and TT homozygous had one band (Figure 1).

### Sequencing

**Figure 2:**
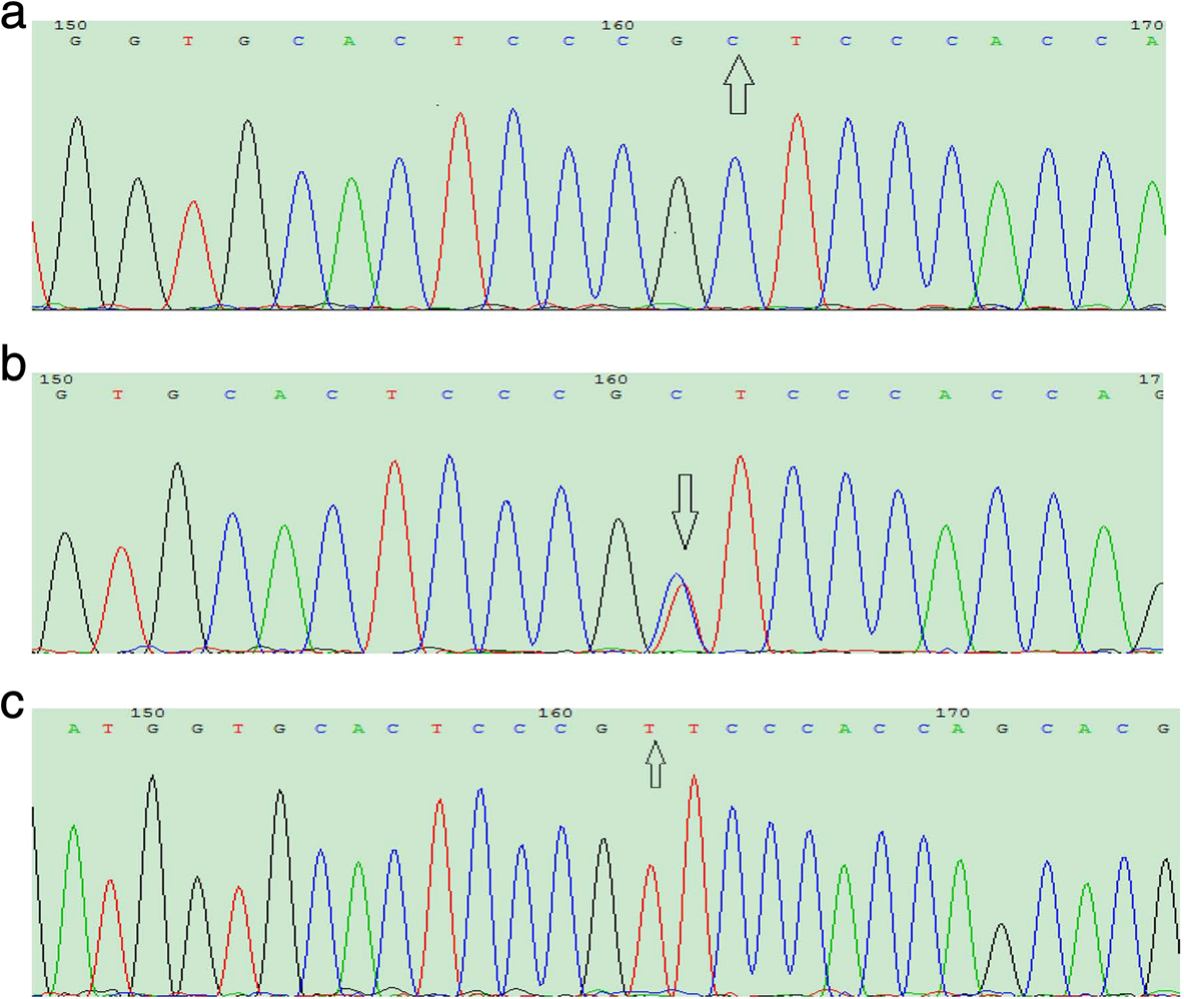
The sequence of CC, CT, and TT genotype, the arrow showed the mutation (a: CC, b: CT, c: TT).

10 of CC type, CT type, and TT type in rs2592551 of GGCX type gene were randomly selected, respectively, for sequencing by Shanghai Sangon Biological Engineering Co., Ltd. The direct sequencing confirmed these genotypes (Figure 2).

### Statistical methods

SPSS17.0 statistical package was used for statistical analysis. The gene counting method was used for allele frequency and genotype frequencies calculation. Each genotype and allele frequencies were calculated, the categorical variables were expressed as percentages. The matching degree of Hardy-Wrinberg genetic equilibrium in our subjects was tested with Chi-square. The warfarin dose between different gender and genotype were compared using rank sum test namely as U test. *P* < 0.05 was considered as statistically significant.

## Abbreviations

TG: Triglycerides; TC: Total cholesterol; HDL-C: High-density lipoprotein; LDL-C: Low-density lipoprotein; GGCX: γ-glutamyl carboxylase; FBG: Fasting blood glucose.

## Competing interests

The authors declare that they have no competing interests.

## Authors’ contributions

XK and M carried out the molecular genetic studies and drafted the manuscript. YCY and WHL carried out the genotyping. ZQL and PYH participated in the design of the study and performed the statistical analysis. WHL conceived of the study, and participated in its design and coordination and helped to draft the manuscript. All authors read and approved the final manuscript.
